# Measurement of cognition and profiling early learning environments in India, Indonesia and Senegal: a UKRI GCRF Action Against Stunting Hub protocol paper

**DOI:** 10.1136/bmjpo-2022-001685

**Published:** 2024-02-27

**Authors:** Julie Dockrell, Jessica Massonnié, Lynn Ang, Bernardita Munoz-Chereau, Sylvia Fernandez Rao, Risatianti Kolopaking, Moustapha Ndiaye, Claire Heffernan

**Affiliations:** 1 Psychology and Human Development, UCL Institute of Education, London, UK; 2 School of Education and Sociology (EDSOC), University of Portsmouth, Portsmouth, UK; 3 UCL, London, UK; 4 Learning and Leadership, UCL, London, UK; 5 Behavioural Science Unit, Extension and Training Division, National Institute of Nutrition, Hyderabad, Telangana, India; 6 Southeast Asian Ministers of Education Organization Regional Center for Food and Nutrition (SEAMEO RECFON)-Pusat Kajian Gizi Regional, Universitas Indonesia, Jakarta, Indonesia; 7 Faculty of Psychology, UIN Syarif Hidayatullah, Jakarta, Indonesia; 8 Cheikh Anta Diop University, Dakar, Senegal; 9 The Royal Veterinary College, UCL, London, UK

**Keywords:** Epidemiology, Growth, Nursing Care, Psychology

## Abstract

**Introduction:**

Childhood stunting is associated with poorer child health, growth and development including diminished cognitive abilities. Mapping out the links between child stunting and Early Childhood Education and Development is critical to increasing understanding of the causes and effects of childhood stunting, and for programme and policy development. The aim of this study is to investigate and compare the development and educational environments across India, Indonesia and Senegal, and to identify the multifactorial drivers and impacts of childhood stunting to inform a new typology.

**Methods and analysis:**

This current study is part of an interdisciplinary observational research study, where women are recruited during pregnancy and mother–infant pairs followed prospectively, up to 24 months after birth. Eight measures will be used to profile children’s early development and learning environments in two sample cohorts: (A) children aged 12 and 24 months born to the women recruited during pregnancy (ie, 500 pregnant mothers per country) and (B) a preschool case–control cohort of siblings from the main cohort aged between 3:6 and 5:6 years of age where anthropomorphic measures will be collected to assess degrees of stunting. Profiling of the development and learning environments in the countries will include both parent/caregiver self-reported and local staff (enumerators) direct assessments of children and settings.

**Ethics and dissemination:**

This study was approved by the institutional ethics committees of all partner institutions. In India, Indian Council of Medical Research-National Institute of Nutrition, Hyderabad; In Indonesia, Ethics Committee of the Faculty of Medicine, University of Indonesia; and in Senegal, National Ethics Committee for Scientific Research in Senegal.The findings of the study will be disseminated in national and international meetings, seminars, conferences and peer-reviewed journals.

**WHAT IS ALREADY KNOWN ON THIS TOPIC**

- Childhood stunting is associated with a range of developmental domains including cognition.
- Stunting co-occurs with challenging life circumstances, making it difficult to ascertain relationships between the impact of poor learning environments, cognition and stunting.
- There is a need to better understand the learning environments of children who are stunted, during and beyond the first 2 years of life.
- Childhood stunting is best considered on a continuum (child height for age Z score).
- Over the last decade, a range of measures for assessing child development in low-income and middle-income countries (LMICs) have been translated, adapted and implemented.

**WHAT THIS STUDY ADDS**

- Enhances the knowledge base of measures of child cognitive development and learning environments for use in LMICs.
- Profiles the quality of learning environments in homes and in early years settings to better understand the links between stunting and child development in a range of settings.

**HOW THIS STUDY MIGHT AFFECT RESEARCH, PRACTICE OR POLICY**

- It enhances children’s educational opportunities by building capacity in the study countries (India, Indonesia and Senegal) to measure early development and learning environments in culturally and contextually appropriate ways.
- It provides a dataset to facilitate international comparisons and interpretation of other data collected within the wider research programme.
- It provides an evidence-based approach to inform programme and policy development oriented to tackle childhood stunting.

## Introduction

Childhood stunting is associated with a range of developmental domains including cognition, language and motor development,^1^ where poorer HAZ (Height-for-Age Z scores) results are associated with lower scores in cognitive, language and motor development measures. Yet, causal pathways of impact are lacking. It is difficult to disentangle the impact of poorer learning environments and childhood growth patterns. Limitations in understanding have resulted from a lack of comparative measures across children, settings and countries over time. Typically measures of cognition are designed for use in English speaking populations in high-income countries with limited attention to cultural and linguistic diversity. Furthermore, little work has focused on assessing the educational and learning environments of children from areas where significant levels of childhood stunting are reported.

The UKRI GCRF Action Against Stunting Hub, with study sites in India, Indonesia and Senegal, aims to apply a whole child approach to disrupt current thinking about the causes and impact of childhood stunting. The goal is to provide an integrated evidence base across the four interlinked ‘environments’ of the child from the physical, home and educational to the wider food environment. The cognition and education work stream underpins this effort by exploring the impact of HAZ on children’s developmental trajectories and the ways in which learning environments can mitigate this impact. This study aims to assess the development and home learning environments of children aged between 12 and 24 months in India, Indonesia and Senegal. Additionally, the study will also profile the early childhood education (ECE) environments attended by children between 3:6 and 5:6 years of age, and further compare the developmental profiles of children’s HAZ in relation to growth limiting exposures.^2^

The development of the protocol for this study is informed by a systematic review of reliable and valid measures of early development and learning environments in low-income and middle-income countries (LMICs),^3^ and by systematic profiling of the ECE systems in India, Indonesia and Senegal. The study will examine the quality of learning environments in each country, the links between growth patterns and child development outcomes and delineate measures to enhance the educational opportunities for children.

## Methods, sample and analysis

This study is part of an interdisciplinary observational study to investigate the multiple factors implicated in child growth and development (child stunting). Prospective pregnancy cohorts were established in Senegal (Kaffrine), India (Hyderabad) and Indonesia (Lombok), where 500 women per country were recruited during pregnancy. The mothers and their infants will be followed up to 24 months of age to understand the contributions of multitude factors such as nutrition, gut health, epigenetics, food environment, food safety, hygiene, cognition, on childhood growth trajectories, where appropriate benchmarks of moderate and severe childhood stunting will be used in the analyses.^4^ The sample size of the main population has been calculated based on statistical power and the frequency of stunting in the cohorts to be studied. The main calculation was based on the epigenetic work (see protocol by Jobarteh *et al*, titled Developing a ‘whole child approach’ to understanding and preventing child stunting: introducing the UKRI GCRF Action Against Stunting Hub). Based on the prevalence of stunting in the target populations (30%–40%), we estimate that our strategy of random population sampling of N=500 would yield the required numbers of 150–200 stunted children per study site.

The cognition and education workstream of the interdisciplinary research programme will focus on understanding early learning environments in the three study sites: India, Indonesia and Senegal. Comparisons will allow the identification of similar and different patterns of relationships to further elucidate the links between poor childhood growth patterns and cognition, language and motor development. The cognition and education research will be divided into two substudies:

### Study 1

This is a cross-sectional study designed to profile developmental skills and learning opportunities of infants at 12–24 months of age. Infants born to women recruited during pregnancy across communities in Senegal (Kaffrine), India (Hyderabad) and Indonesia (Lombok) will be enrolled in this study.

#### Data collection procedure

This study will involve direct assessment of children at 12 and 24 months of age by both local staff (enumerators) and parents/caregivers reports. The study will use specific direct child assessments and questionnaires to assess the different components of early child development. The measures include: (A) Oxford Neurodevelopment Assessment (Ox-NDA) (12 months)—the OX-NDA comprises 41 items measuring children’s cognition, motricity, language, executive functions, positive behaviour and negative behaviour.^5 6^ The measure is designed to be used by trained enumerators, but no formal qualifications are required. (B) Inter-NDA (24 months)—the International Fetal and Newborn Growth Consortium for the 21st century Neurodevelopmental Assessment (Inter-NDA) comprises 39 items measuring cognition, motricity, language, positive behaviour and negative behaviour.^7–10^ The measure is designed to be used by trained enumerators, but no formal qualifications are required.

1. Bayley IV Cognition scale (12 and 24 months)—Following the test standardisation baseline measures will be established and children will progress through the assessment until they reach the discontinuation point t (starting and stopping rules are specified in the manual).^11^ This scale will be administered by appropriately qualified psychologists in each setting.
2. HOME inventory (12 and 24 months)—the infant and toddler HOME version used in previous studies examining the impact of stunting in LMIC (eg, the MAL-ED study)^12–14^ will be used. The HOME version comprises 48 items and measures emotional and verbal responsivity from the caregiver, avoidance of restriction and punishment, promotion of child development by the caregiver, organisation of the physical and temporal environment, provision of appropriate play material, opportunities for variety in daily stimulation and cleanliness of child. The observation is scheduled at a time when the primary caregiver and the child are at home. All enumerators will be trained in the use of the HOME inventory.

The homes of study participants will be visited by local trained staff in each country and the questionnaire administered following a standard operating protocol for training and administration. The OX-NDA will be administered at twelve months of age and Inter-NDA will be administer at 24 months of age (ie, 1 year later after OX-NDA). At both time points (12 and 24 months), observations of the home environment will be carried out concurrently using the HOME inventory, and 50 children (10% of the sample in each country) will be administered the Bayley IV cognition scale to cross-validate the Ox-NDA and Inter-NDA measures. To calculate the sample for the Bayley IV, we followed the results of Bridges and Holler^15^ where data indicated that studies ought optimally to use 50–75 participants per cell. Our target is a minimum of 50 participant per cell (country by age group) with a total sample of 300 children for benchmarking.

Reliability and validity of the measurements will be calculated and the data for each measure presented. Table 1 reports validity and reliability of each measure obtained in previous relevant studies in LMIC.^3^ Hence table 1 is not exhaustive, but indicative of the evidence base for the inclusion of the measures.

**Table 1:** Comparison of the age range, scoring procedures, administration time, reliability, validity and cross-cultural use of the measures obtained in previous studies and included in study 1

	Age range	Scoring	Administration time	Reliability	Validity	Example of use in LMICs
Ox-NDA	10– 14 months	Four-point Likert scale or three-point Likert scale, based on direct observation	20–30 min	Inter-rater reliability (k=0.80-.96) Test–retest reliability (k=0.85–0.94) Internal consistency >0.70 for all subscales except language (α=0.40) and negative behaviour (α=0.22)	Moderate agreement between cognitive and motor domains of the OX-NDA and Bayley-III (ICCs: 0.63 and 0.68); satisfactory agreement for language (ICC: 0.30)	Brazil
Inter-NDA	22–30 months	Four-point Likert scale or three-point Likert scale, based on direct observation or caregiver report	15 min	Inter-rater reliability (k=0.70) Test–retest reliability (k=0.79) Internal consistency: α>0.80 (cognition, language, positive behaviour); α=0.56 (negative behaviour)	Tested against Bayley III-better sensitivity and specificity for cognitive scale vs language scale	Brazil, India and Kenya
Bayley IV	16 days to 24 months	Polytomous (not present, emerging, mastery) based on direct observation	Full scale: 50 min at 12 months 84 min at 24 months	Cognitive Scale: test–retest (r=82) Internal consistency (split-half): r=0.95	Less than 5-point discrepancy with WPPSI-IV and PDMS2	Bayley III has been used in Tanzania, Brazil, Malaysia and Vietnam
HOME	0–3	Dichotomous (yes, no) based on the observation of the home environment, on caregiver’s report or either of them	60 min	After removing Avoidance of Punishment, three factors can be extracted (Emotional and Verbal Responsivity; Environmental Safety, Child Cleanliness) all with α >0.70	Environmental Safety and Child Cleanliness correlate with crowding in the home	Columbia, Bangladesh, Mexico, Pakistan, Jamaica, India, Nepal, Peru, Tanzania, Brazil and South Africa

Bayley IV, Bayley Scales of Infant Development IV; ICC, Intraclass Correlation Coefficient; Inter-NDA, International Fetal and Newborn Growth Consortium for the 21st Century Neurodevelopmental Assessment; LMICs, low-income and middle-income countries; OX-NDA, Oxford Neurodevelopmental Assessment at twelve months of age; PDMS-2, Primary Scale of Intelligence Second Edition; WPPSI-IV, Wechsler Preschool and Primary Scale of Intelligence Fourth Edition.

The rationale for choosing the measures was previously reported in a systematic literature review conducted at the beginning of the project.^3^ Through that review we found suitable measures based on psychometric quality (validity and reliability), cultural adaptability and use in the field sites. Although we originally chose Bayley Scales of Infant Development III (BSID-3), we decided to use BSID-4. This is a newer version of the BSID-3 capturing development in a more nuanced way, as items can be scored for mastery, emerging or not present. It will be administered by experienced psychologists and overall, it will provide local teams with the experience and skills to apply it in the future.

#### Interpretation of the measures

A systematic process of translation, back translation and cultural adaptation will be followed for all measures to ensure that each questionnaire is valid and culturally appropriate for the study context.^4^ The measures will be translated from English to the languages spoken at the study sites before use (India: Telugu and Hindi; Indonesia: Bahasa, Sasak; Senegal: French and Wolof). A panel of bilingual individuals will be convened to resolve expressions or concepts, which may appear vague (ie, either not understood or not clear) to ensure accurate translation of each question. Measures will then be back translated. All measures will be piloted in local settings. Regular meetings will take place with the education and cognition team members to ensure that the content, scoring options and materials depicted in the items are appropriate. Pilot data will be discussed, and onsite training will occur before the start of the main assessment period.

### Study 2

This second substudy will compare the learning needs and educational opportunities of a preschool cohort of children aged between 3:6 and 5:6 years old using a case–control study design. The study will recruit older siblings of children who meet the age criterion across communities in Senegal (Kaffrine), India (Hyderabad) and Indonesia (Lombok). Measurements of childhood growth will be collected using standardised anthropomorphic measures. As with study 1 both a continuum of HAZ measures and cut-offs will be used to profile the population. This study will be conducted in two phases to provide a comprehensive understanding of the existing early years education setting and its link with child growth and development.

#### Phase 1: profiling early learning environments

A representative sample of early years settings will be selected in each study site following a systematic four-step process. First, a documentary analysis will be used to establish the types of learning settings that exist in each study site and the proportion of children attending the settings. Second, the number and types of settings in each study site will be referenced. Third, stratification variables will be outlined, including: the types of settings, source of funding, targeted age group, locality (urban or rural), school size, class size and the profile of children attending the setting such as the socioeconomic background, gender, ethnicity and religion.^16–24^ Fourth, a sample of settings will be selected in each study site according to the stratification variables. The selection will include at least 20% of the existing settings in the study site. In districts where there are 20 or fewer educational settings all will be invited to participate in the study. The settings included in the sampling plan will be visited in 2022.

#### Phase 2: comparisons of the learning needs and the environments of siblings

Households of women recruited during pregnancy in the observational study will be contacted for identification of households with children 3:6–5:6 years of age. Mothers in eligible household will be asked if the identified child is enrolled in a preschool and the name of the preschool. For children in preschool, these settings will be contacted for participation in the study. For children not in preschools child and learning environment measures will be collected in the home. Children’s cognitive performance will be profiled using the International Development and Early Learning Assessment (IDELA) as a robust and reliable global tool that measures children’s early learning and development (ages 3:5–6:0).^25–28^

Power calculation for inclusion of children was performed to determine the minimum sample size needed to detect a small effect size (f=0.1) in the analysis of the association between height and child development (education and cognition) as reported in previous studies.^29–32^ The sample size was calculated at a power of 80% and an error probability of 5%, considering the inclusion of seven covariates (age, gender, disability, family size, parental education, parental income and preschool attendance). The sample size calculations using regression models with height as a continuous variable and analysis of covariance comparing stunted and not stunted children yielded a minimum sample size of 787 children. Thus, a target of 300 children will be recruited per country, a total of 900 children for the full study.

#### Data collection procedure

1. Local staff (enumerators) will be trained on all measures and piloting will take place with the enumerators. The early years settings will be visited during a normal preschool day to profile the learning environment. The days and times of observation will be randomly selected for each preschool using the preschool’s schedule. In phase 1, the Measuring Early Learning Environment (MELE) will be used to profile ECE, and teachers will be interviewed using a qualitative teacher interview (QTI). In phase 2, children’s profiles of development will be added and where children are not in ECEs the home learning environment will be profiled. MELE—contains 52 items grouped in 5 sections/grids: (1) basic classroom observation (eg, number of children present), (2) learning activities, (3) classroom interactions and approaches to learning, (4) classroom arrangement, space and materials, and (5) facilities and safety.^17–20^ The MELE grid will be used to profile early learning environment in phase 1 of study 2.
2. IDELA is composed of 24 items measuring socioemotional development, motor development, emergent language and literacy, emergent maths and numeracy, executive functions and children’s approaches to learning.^25–28^ The enumerators will administer IDELA in phase 2 of the study. Assessment will be conducted with children at the preschool setting or in home environment (for children who do not attend an ECE setting).
3. HOME early childhood scale. The HOME is a descriptive profile that leads to an assessment of a child’s home environment. It measures the amount and quality of support and stimulation available to the child at home. Eight domains are measured: learning materials, language stimulation, physical environment, responsivity, academic stimulation, modelling, variety and acceptance. The scale will be adapted to each local context and piloted to ensure cultural appropriateness of the items.
4. QTI—elicits teachers’ voices through a conversation about five key dimensions: (1) context, (2) teacher experience, (3) professional development, (4) learning opportunities and needs and (5) quality of early learning environments. These dimensions have been previously identified as relevant when exploring quality learning environments in LMICs.^16–20^
  The QTI will contextualise the MELE, IDELA and HOME early childhood scale data, as well as inform a Toolkit, which aims to further (1) support the professional development of teachers to enhance children’s learning; (2) enhance the quality of classroom interactions and the environment that can best support learning for children who are stunted and (3) gain a better understanding of the complexities of characterising quality early years settings.

The reliability and validity of the measurements will be calculated and presented. The characteristics of the methods and their reliability and validity reported in previous studies are presented in table 2. The rationale for choosing the measures was previously reported in a systematic literature review conducted at the beginning of the project. Through that review we found suitable measures based on psychometric quality (validity and reliability), cultural adaptability^3^ and use in the field sites.

**Table 2:** Comparison of the age range, scoring procedures, administration time, reliability, validity and cross-cultural use of the measures obtained in previous studies and included in study 2

	Age range	Scoring	Administration time	Reliability	Validity	Use in LMICs
IDELA	3:6– 6:6	Dichotomous (correct, incorrect), continuous or Likert scales, based on direct observation	35 min	Inter-rater reliability (0.70–0.99) Internal consistency (0.66–0.95)	Convergent validity: 0.33–0.61 Internal consistency: 0.66–0.95	India and Indonesia
MELE	Classroom-based observation	Dichotomous, continuous, Likert scales or predefined answer options	120 min	Three factors: health and safety (α=0.65), materials and activities (α=0.87), teacher–child interactions (α=0.83)	Validated against policy regulations and curriculum for ECE	Tanzania
HOME (early childhood scale)	3:6–5:6	Dichotomous (yes, no), based on direct observation	45–60 min	Inter-rater reliability (.70-.90)	Validity: 0.7	Bangladesh, Colombia and Mexico

ECE, early childhood education; HOME, Home Observation for Measurement of the Environment; IDELA, International Development and Early Learning Assessment; LMICs, low-income and middle-income countries; MELE, Measuring Early Learning Environment.

#### Interpretation of the measures

A systematic method of translation and adaptation of the MELE, IDELA, HOME and QTI will be followed as previously described herein. Furthermore, the content of MELE will be validated with external stakeholders involved in the implementation, administration and monitoring of ECE. Once the adapted measures are finalised, a back translation will be conducted. Discrepancies between the back-translated English version and the original English version should adapt to the cultural context and target population. However, the discrepancies will be revised if it is considered to depart from the intended meaning of the item.

### Analysis plan

We will conduct both descriptive and inferential statistical analyses. Given the large data sets and working across countries data cleaning and verification will be conducted by site. The first analyses will be done by country site and then, if appropriate conceptually and statistically, with comparisons across the three different sites to examine similarities and differences in patterns of relationships. Descriptive statistics will provide means (SDs) for child level IDELA and HOME by gender and age in months. MELE delivers a profile of scores to capture the learning environments. First, we will establish the profiles of early years provision across the study sites. This will provide a comparative benchmark with international data and for the ECE settings for the sibling cohort.^17 18^

We plan three separate approaches to the analyses of child data. In the first instance, we will use HAZ as a continuous variable in regression models to examine associations with developmental profiles controlling as appropriate for education settings. These analyses test the prediction that childhood preschool cognition is impacted by HAZ, controlling for learning environment, age and gender. In a second set of analyses, we will use the WHO stunting criteria to compare children who are stunted and children who are not stunted. Finally, in order to account for the hierarchical nested nature of our data, we will explore relationships between the sibling’s growth and cognition using multilevel modelling.

We will also use qualitative analysis to explore teachers’ perceptions regarding positive educational environments. We will conduct QTI with teachers and analyse them through thematic analysis. QTIs will be conducted face to face in the preschool settings, audiorecorded and transcribed. We will analyse the interview transcripts by developing a hybrid method to content analysis,^33^ which combines a deductive top-down approach (that explores the presence of themes informed by categories drawn from previous knowledge of the literature and themes derived from our research questions) with an inductive bottom-up approach (what participants said). The deductive coding will consist of interrogating the data using categories informed by the interview schedule (such as ‘context’ and ‘teacher experience’).

More precisely, the deductive coding will explore the following aspects presented in table 3.

**Table 3:** Codes to be used in the deductive coding of QTI

QTI section/categories	QTI questions	Codes
Context	What is the age range of the children enrolled in your classroom?	Age range
	What proportion of children are aged 3–5?	Proportion
	Is children’s height and weight measured? If yes: How is it measured? How often? How is it stored? If no: Do you access to these measures? Who provides these measures?	Height and weight
	Is food served at the programme (either a meal or snack provided by the programme or children bring from home)? If yes: What meals or snacks are served? (Snack served only, Lunch served only, Snacks and lunch are served), Who provides the food? (The programme provides all food, The programme provides some food and parents provide some, Parents provide all food), Is there a daily menu? In a normal week, how many days is a meal served? (None, 1–3 4+), If a meal is served less than every day, please indicate the reason why: Holiday, Ration not available, Other (specify)	Food
	Is water served at the programme? If yes: Where does the water come from? Who provides the water? How do children drink it? (ie, cups, bottles)	Water
	How COVID has affected the organisation of learning (ie, conditions, who is attending)?	Covid
Teacher experience	What is your main role in the (centre/setting)?	Main role
	How many years have you worked in this pre primary centre?	Years in the setting
	How many years have you been a teacher overall (for any grade)?	Overall
	What is the highest qualification level you have completed?	Highest qualification
	Did you need to complete a qualification or training to become a teacher? If yes: What was it?	Requirements
	Why did you choose to become an early years teacher?	Reason
	If you could turn back time, would you choose again to become a teacher? Why?	Choice
Professional development	What training opportunities are there in your setting?	Professional opportunities
Professional development	If you were offered training, what would you find useful?	Useful training
Children learning experiences and opportunities	Please describe a normal working day with the children	Routine
Children learning experiences and opportunities	What activities are routinely provided?	Activities
	What do you think children enjoy most about coming here?	Enjoy
	What do you see as most important in children’s education?	Priority
	When a child starts pre-school here, what can they normally do on their own?	Can do
	How do you know if a child needs more support with their learning?	Support identified
	How do you support their learning?	Support provided
	Are there any targets or goals that you use to guide teaching and learning? If yes: What are these targets? Who defines these targets? Are they evaluated? How?	Targets
	Do you keep children’s portfolios? If yes: How does it work? Who decides what goes on the portfolio? Who has access to the portfolio?	Portfolios
Quality early learning environment	According to your view, what are the main opportunities/strengths in the learning environment you work in?	Opportunities
	What support would improve children’s learning opportunities? (ie, more materials, more training, community support)	Needs
	When you think about the best preschool: How is teaching delivered? How the environment looks like? how is it the space set up? What materials are available? What interactions are promoted?	Ideal scenario
	How would you describe the main similarities between these pictures and your setting?	Similarities
	How would you describe the main differences between these pictures and your setting?	Differences
Emergent	Is there anything else you would like to add?	Emergent

QTI, qualitative teacher interview.

The deductive coding will be followed by inductive coding where we will assign meaning to segments of text using categories (eg, ‘challenges’ or ‘support’) that might appear in the interview transcripts. Both coding procedures will break down the transcriptions into smaller pieces of information and compare the pieces for similarities and differences before regrouping them under themes and categories.^34^ We will create coding schemes using Excel spreadsheets. Interviews will be analysed into one coding scheme per country. We will implement intracase analysis (within country), followed by intercase analysis (between countries).^35^ We will do this by taking an iterative process of categorising and connecting data in order to understand how the data relate and interact within and across contexts.

The QTI thematic analysis will be integrated with other measures (MELE and IDELA) to gain a better understanding of (A) the quality of early years settings from the perspectives of teachers and adults involved in supporting children’s learning and (B) the specific cultural context of what makes a positive learning environment according to each setting.

### Patient and public partnership strategy

The study proposal, including the methods used for assessing early learning and environment, was discussed with key stakeholders involved in the delivery of early learning curriculum in India, Indonesia and Senegal. Community engagement meetings were organised in the study communities to discuss the design, its benefits and the interpretation of its outcomes. Questions and answer sessions were delivered in the engagement plan to ensure questions are answered and there is adequate clarity around the purpose/intention of the research study. The study participants and public will be involved in the dissemination of the study findings.

## Ethics and dissemination

As a research team, we are committed to the principles of fair, equitable and ethical research partnerships and ensuring that the research is undertaken to the highest professional standards of ethics and integrity. This study was approved by the institutional ethics committees of all partner institutions. In India, Indian Council of Medical Research-National Institute of Nutrition, Hyderabad; In Indonesia, Ethics Committee of the Faculty of Medicine, University of Indonesia; and in Senegal, National Ethics Committee for Scientific Research in Senegal. The research will be grounded in the ethical principles of confidentiality, informed consent, risk mitigation and ‘do no harm.’ We are guided by the professional code of conduct specific to each discipline as set out in the British Educational Research Association and British Psychological Society ethical guidelines. The findings of the study will be presented in national and international meetings, seminars, conferences and published in peer-reviewed journals. The study’s data will be deposited in a public data repository.

## Supplementary Material

Author's manuscript

## Data Availability

Data are available on reasonable request.
