# Supplementary material for: Measurement of cognition and profiling early learning environments in India, Indonesia and Senegal: a UKRI GCRF Action Against Stunting Hub protocol paper
Source: BMJ Paediatr Open. 2024 Feb 27;8(Suppl 1):e001685. doi: 10.1136/bmjpo-2022-001685 (PMC10900338; doi:10.1136/bmjpo-2022-001685)
Supplement: Author's manuscript [file bmjpo-2022-001685.draft_revisions.pdf]

BMJ Paediatrics Open

BMJ Paediatrics Open is committed to open peer review. As part of this commitment we make the peer review history of every article we publish publicly available.

When an article is published we post the peer reviewers' comments and the authors' responses online. We also post the versions of the paper that were used during peer review. These are the versions that the peer review comments apply to.

The versions of the paper that follow are the versions that were submitted during the peer review process. They are not the versions of record or the final published versions. They should not be cited or distributed as the published version of this manuscript.

BMJ Paediatrics Open is an open access journal and the full, final, typeset and author-corrected version of record of the manuscript is available on our site with no access controls, subscription charges or pay-per-view fees (http://bmjpaedsopen.bmj.com).

If you have any questions on BMJ Paediatrics Open's open peer review process please email info.bmjpo@bmj.com

BMJ Paediatrics Open

Measurment of cognition and profiling early learning environments in India, Indonesia and Senegal: a UKRI GCRF Action Against Stunting Hub protocol paper

Journal:	*BMJ Paediatrics Open*
Manuscript ID	bmjpo-2022-001685
Article Type:	Protocol
Date Submitted by the Author:	26-Sep-2022
Complete List of Authors:	Dockrell, Julie; UCL<br>Massonie, Jessica; University of Portsmouth<br>Ang, Lynn; UCL<br>Munoz-Chereau, Bernardita; UCL, Learning and Leadership<br>Fernandez Rao, Sylvia; National Institute of Nutrition<br>Kolopaking, Risatianti; UIN Syarif Hidayatullah Jakarta<br>Ndiaye, Moustapha; Cheikh Anta Diop University of Dakar<br>Heffernan, Claire; UCL
Keywords:	Epidemiology, Growth, Nursing Care, Psychology

SCHOLARONE™
Manuscripts

I, the Submitting Author has the right to grant and does grant on behalf of all authors of the Work (as defined in the below author licence), an exclusive licence and/or a non-exclusive licence for contributions from authors who are: i) UK Crown employees; ii) where BMJ has agreed a CC-BY licence shall apply, and/or iii) in accordance with the terms applicable for US Federal Government officers or employees acting as part of their official duties; on a worldwide, perpetual, irrevocable, royalty-free basis to BMJ Publishing Group Ltd ("BMJ") its licensees and where the relevant Journal is co-owned by BMJ to the co-owners of the Journal, to publish the Work in this journal and any other BMJ products and to exploit all rights, as set out in our licence.

The Submitting Author accepts and understands that any supply made under these terms is made by BMJ to the Submitting Author unless you are acting as an employee on behalf of your employer or a postgraduate student of an affiliated institution which is paying any applicable article publishing charge ("APC") for Open Access articles. Where the Submitting Author wishes to make the Work available on an Open Access basis (and intends to pay the relevant APC), the terms of reuse of such Open Access shall be governed by a Creative Commons licence – details of these licences and which Creative Commons licence will apply to this Work are set out in our licence referred to above.

Other than as permitted in any relevant BMJ Author's Self Archiving Policies, I confirm this Work has not been accepted for publication elsewhere, is not being considered for publication elsewhere and does not duplicate material already published. I confirm all authors consent to publication of this Work and authorise the granting of this licence.

Confidential: For Review Only

Measurement of cognition and profiling early learning environments in India, Indonesia and Senegal: a UKRI GCRF Action Against Stunting Hub protocol paper

Authors: Dockrell, Julie[1]; Massonie, Jessica[2]; Ang, Lynn[3]; Munoz-Chereau, Bernardita[4]; Fernandez Rao, Sylvia[5]; Kolopaking, Risatianti[6]; Ndiaye, Moustapha[7]; Heffernan, Claire[8]

Affliations: IOE, UCL's Faculty of Education and Society, UK [1 3 4]; University of Portsmouth, School of Education and Sociology (EDSOC)[2;] Behavioural Science Unit, Extension and Training Division, ICMR-National Institute of Nutrition, India[5]; London International Development Centre (LIDC) London School of Hygiene and Tropical Medicine, UCL Institute of Education, UK[8;] UIN Syarif Hidayatullah Jakarta, Indonesia[6]; Cheikh Anta Diop University Dakar, Senegal[7]

Corresponding author: Munoz-Chereau, Bernardita

Email: b.munozchereau@ucl.ac.uk

Key words: Cognition, Learning, Early Childhood Education, Children, Reducing Stunting, Early Development

Confidential: For Review Only

ABSTRACT

Introduction

Child stunting has a dampening impact on child health, growth and development including dimished cognitive abilities. Mapping out the links between child stunting and Early Childhood Education and Development (ECED) is critical to increasing understanding of the causes and effects of child stunting, and for programme and policy development. The aim of this study is to investigate and compare the development and educational environments across India, Indonesia and Senegal, and to identify the multifactorial drivers and impacts of child stunting to inform a new typology.

Methods and analysis

This study is part of an interdisciplinary observational research, where women are recruited during pregnancy and mother-infant pairs followed prospectively, up to 24 months after birth. The study will use eight measures to profile children's early development and learning environments in two sample cohorts: a) children aged 12 and 24 months born to the women recruited during pregnancy (i.e., 500 pregnant mothers per country), and b) a preschool case-control cohort of siblings from the main cohort aged between 3:6 to 5:6 years of age who are stunted and not stunted. Profiling of the development and learning environments in the countries will include both parent/caregiver self-reported and local staff (enumerators) observed assessments.

Ethics and Dessimination

This study was approved by the institutional ethics committees of LSHTM, UCL Institute of Education and local country partner institutions in India, Indonesia and Senegal. The findings of the study will be presented in national and internation meetings, seminars, conferences and published in peer-reviewed journal.

Confidential: For Review Only

SUMMARY BOX

What is known about the subject	What this study hopes to add
• Child stunting impacts a range of developmental domains including congition.<br>• Stunting co-occurs with challenging life circumstances, making it difficult to disentangle the impact of poor learning environments and stunting.<br>• There is a need to better understand the learning environments of children who are stunted, during and beyond the first two years of life.	• To identify, translate and adapt measures of child cognitive development and learning environments for use in low- and middle-income countries.<br>• To profile the quality of learning environments, and better understand the links between stunting and child development in a range of settings.<br>• To build capacity in the study countries (India, Indonesia and Senegal) to measure early development and learning environments in culturally and contextually appropriate ways, to ultimately enhance children's educational opportunities.

Confidential: For Review Only

INTRODUCTION

Child stunting impacts a range of developmental domains including cognition, language, and motor development (Perkins et al., 2017). Yet, causal pathways of impact are lacking. It is difficult to disentangle the impact of poor learning environments and stunting per se. Limitations in understanding have resulted from a lack of comparative measures across children, settings and countries over time. Typically measures of cognition are designed for use in English speaking populations in high-income countries with little attention to cultural and linguistic diversity. Furthermore, little work has been done assessing the educational and learning environments of children who are stunted.

The UKRI GCRF Action Against Stunting Hub, with study sites in India, Indonesia and Senegal, aims to apply a whole child approach to disrupt current thinking about the causes and impact of child stunting. The goal is to provide an integrated evidence base across the four inter-linked 'environments' of the child from the physical, home and educational to the wider food environment. The cognition and education work stream underpins this work by exploring the impact of stunting on children's developmental trajectories and the ways in which learning environments can mitigate this impact. This study aims to assess the development and home learning environments of children aged between 12 and 24 months in India, Indonesia and Senegal. Additionally, the study will also profile the early childhood education (ECE) environments attended by children between 3:6 and 5:6 years of age, and further compare the developmental profiles of children who are stunted and not stunted.

The development of the protocol for this study is informed by a systematic review of reliable and valid measures of early development and learning environments in low- and middle-income countries (Munoz-Chereau et al., 2021), and by systematic profiling of the ECE systems in India, Indonesia and Senegal. The study will examine the quality of learning

Confidential: For Review Only

environments in each country, the links between stunting and child development outcomes, and delineate measures to enhance the educational opportunities for children.

Confidential: For Review Only

METHODS AND ANALYSIS

This study is part of an interdisciplinary observational study to investigate the multifactors implicated in the ameriolation of child growth and development (child stunting). Prospective pregnancy cohorts were established in Senegal (Kaffrine), India (Hyderabad) and Indonesia (Lombok), where 500 women per country were recruited during pregnancy. The mothers and their infants followed up to 24 months of age to understand the contributions of multitude of factors such as nutrition, gut health, epigenetics, food environment, food safety, hygiene, cognition, etc., in child stunting. The cognition and education workstream of the interdisciplinary research will focus on understanding early learning environments in India, Indonesia and Senegal and further elucidate the links between child stunting and cognition, language and motor development. The congition and education research will be divided into two sub-studies:

Study 1

This is a cross-sectional study designed to compare the learning needs and educational opportunities of infants at 12 – 24 months of age. Infants born to women recruited during pregnancy across communities in Senegal (Kaffrine), India (Hyderabad) and Indonesia (Lombok) will be enrolled in this study.

Data collection procedure

This study will involve direct assessment of children at 12 and 24 months of age by both local staff (enumerators) and parents/caregivers. The study will use specific questionnaires to assess the different components of early child develpment, including:

a) **Ox-NDA (12 months)**– the Oxford Neurodevelopment Assessment (OX-NDA) questionnaire comprises of forty-one items measuring children's cognition, motricity, language, executive functions, positive behaviour and negative behaviour (Fernandes, 2019; Fernandes, under review).

Confidential: For Review Only

b) Inter-NDA (24 months)– the Internationl Fetal and Newborn Growth Consortium for the 21st century Neurodevelopmental Assessment (Inter-NDA) questionnaire comprises of thirty-nine items measuring cognition, motricity, language, positive behaviour, and negative behaviour (International Fetal and Newborn Growth Consortium for the 21st Century Study Group, [INTERGROWTH-21st], 2014; Fernandez et al., 2014; Fernandes, 2020, Villar et al., 2019).

c) Bayley IV Cognition scale (12 and 24 months) – the exact number of items administered to each child depends on their age and performance at the assessment (starting and stopping rules are specified in the manual, Bayley & Aylward, 2019).

d) HOME inventory (12 and 24 months)– the infant and toddler HOME version used in previous studies within similar environment (e.g., the MAL-ED study which was adapted from Black (2004)) (Jones et al., 2017; Murray-Kolb et al., 2014) was selected for inclusion in this study. The HOME version comprises of forty-eight items and measures emotional and verbal responsivity from the caregiver, avoidance of restriction and punishment, promotion of child development by the caregiver, organisation of the physical and temporal environment, provision of appropriate play material, opportunities for variety in daily stimulation, cleanliness of child. The observation is scheduled at a time when the primary caregiver and the child are at home.

The homes of study particiants will be visited by local trained staff in each country and the questionnaire administed following a systematic plan. The OX-NDA will be administed at twelve months of age and Inter-NDA will be administer at twenty-four months of age (i.e., one year later after OX-NDA). At both time points (12 and 24 months), observations of the home environment will be carried out concurrently using the HOME inventory, and fifty children (10% of the sample in each country) will be administered the Bayley IV cognition scale to cross-validate the Ox-NDA and Inter-NDA measures. Reliability and validity of the

Confidential: For Review Only

measurements will be calculated and the data for each measure presented. Table 1 shows the acceptable range of the validity and reliability of each questionniare.

Interpretation of the questionnires

A systematic process of translation and adaptation will be followed to ensure that each questionnaire is valid and culturally appropriate for the study context (Fernald, 2017). The questionnaires will be translated from English to the languages spoken at the study sites before use (India: Telegu and Hindi; Indonesia: Bahasa, Sasak; Senegal: French and Wolof). A panel of bilingual individuals will be convened to resolve expressions or concepts which may apper vague (i.e., either not understood or not clear) to ensure accurate translation of each question. Meetings will be conducted with the Education and Cognition team members to ensure that the content, scoring options and materials depicted in the items are culturally appropriate. Children's books to be used for the literacy items will be selected based on what is typically available in each country's ECE settings. The aim for the material is to reflect local learning conditions.

Table 1.

Comparison of the age range, scoring procedures, administration time, reliability, validity and cross-cultural use of the measures selected for inclusion in Study 1.

	**Age range**	**Scoring**	**Administration time**	**Reliability**	**Validity**	**Use in LMICs**
**Ox-NDA**	10 to 14 months	Four-point Likert scale or three-points Likert scale, based on direct observation	20-30 minutes	Inter-rater reliability (k = .80-.96) Test-retest reliability (k = .85-.94)<br><br>Internal consistency >.70 for all subscales except language (α =	Moderate agreement between cognitive and motor domains of the OX-NDA and Bayley-III (ICCs: 0.63 and 0.68); satisfactory agreement for	Brazil

Confidential: For Review Only

				.40) and negative behaviour (α = .22)	language (ICC: 0.30)	
**Inter-NDA**	22 to 30 months	Four-point Likert scale or three-points Likert scale, based on direct observation or caregiver report	15 minutes	Inter-rater reliability (k = .70) Test-retest reliability (k= .79)<br><br>Internal consistency: α > .80 (cognition, language, positive behaviour); α = .56 (negative behaviour)	Tested against Bayley III – better sensitivity and specificity for cognitive scale vs language scale	Brazil, India and Kenya
**Bayley IV**	16 days to 24 months	Polytomous (not present, emerging, mastery) based on direct observation	Full scale: 50 min at 12 months 84 minutes at 24 months	Cognitive Scale: Test-Retest (*r* = 82) Internal consistency (split-half): *r* = .95	Less than 5-points discrepancy with WPPSI-IV and PDMS2	Bayley III has been used in Tanzania, Brazil, Malaysia and Vietnam
**HOME**	0 to 3	Dichotomous (Yes, No) based on the observation of the home environment, on caregiver's report, or either of them	60 minutes	After removing Avoidance of Punishment, three factors can be extracted (Emotional and Verbal Responsivity; Environmental Safety, Child Cleanliness) all with α > .70	Environmental Safety and Child Cleanliness correlate with crowding in the home.=	Columbia, Bangladesh, Mexico, Pakistan, Jamaica, India, Nepal, Peru, Tanzania, Brazil and South Africa

Notes. Bayley IV: Bayley Scales of Infant Development IV; Inter-NDA: International Fetal and Newborn Growth Consortium for the 21st Century Neurodevelopmental Assessment; OX-NDA: Oxford Neurodevelopmental Assessment at twelve months of age; PDMS-2: Primary Scale of Intelligence Second Edition; WPPSI-IV: Wechsler Preschool and Primary Scale of Intelligence Fourth Edition

Confidential: For Review Only

Study 2

This study will compare the learning needs and educational opportunities of a preschool cohort of children aged between 3:6 and 5:6 years old using a case-control study design. The study will recruit siblings of children who meet the age criterion across communities in Senegal (Kaffrine), India (Hyderabad) and Indonesia (Lombok). Measurements of childhood stunting will be collected. (children who are stunted (i.e., Length for Age (LAZ) Z-score ≥ -2 SD (standard deviation) of the WHO growth standard) and not stunted). This study will be conducted in two phases to provide a comprehensive understanding of the existing early years setting and its link with child growth and development.

Phase 1: Profiling Early Learning Environments

A representative sample of early years settings will be selected in each study site following a four-steps process. Firstly, a documentary analysis will be used to establish the types of learning settings that exist in each study site and the proportion of children attending the settings. Secondly, the number and types of settings in each study site will be referenced. Thirdly, stratification variables will be outlined, including: the types of settings, source of funding, targeted age group, locality (urban or rural), school size, class size, and the profile of children attending the setting such as the socio-economic background, gender, ethnicity and religion (Iskandar, 2020, Rao, 2021; Salmon & Dramani, 2015, Seurat, 2016, Singh & Mukherjee, 2017, UNICEF, 2019, World Bank, 2020). Fourthly, a sample of settings will be selected in each study site according to the stratification variables. The selection will include at least twenty percent of the existing settings in the study site. The settings included in the sampling plan will be visited in early 2022.

Phase 2: Comparisons of the learning needs and environments of stunted and not stunted siblings

Confidential: For Review Only

Household surveys will be used to identify eligible households. Households of women recruited during pregnancy in the observational study will be contacted for identification of households with children aged 3:6- to 5:6 years of age . Mothers in eligible household will be asked if the identified child is enrolled in a school and the name of the school. For children in school these settings will be contacted for participation in the study. For children not in schools child and learning environment measures will be collected in the home.

Power calculation for inclusion of children was performed to determine the minimum sample size needed to detect a small effect size (f = .1) in the analysis of the association between height and child development (education and congition) as reported in previous studies (Nasir et al., 2012, Nurliyana et al., 2020; Scharf et al., 2018). The sample size was calculated at a power of 80% and an error probability of 5% taking into account the inclusion of seven covariates (age, gender, disability, family size, parental education, parental income, preschool attendance). The sample size calculations using regression models with height as a continuous variable and Analysis of Covariance (ANCOVA) comparing stunted and not stunted children yielded a minimum sample size of 787 children. Thus, a target of 300 children will be recruited per country, a total of 900 children.

Data collection procedure

Local staff (enumerators) trained in the assessments methods will visit the early years settings during a normal school day to observe the learning environment. The days and times of observation will be randomly selected for each school using the school's schedule. Three instruments will be used to assess the learning environment in both pases (phase 1 and 2) of the study.

a) Measuring Early Learning Environment (MELE) – it contains fifty-two items grouped in five sections/grids: (i) basic classroom observation (e.g. number of children present), (ii) learning activities, (iii) classroom interactions and approaches to learning, (iv) classroom arrangement,

Confidential: For Review Only

space and materials, (v) facilities and safety (Raikes et al., 2019, 2020). The MELE grid will be used to profile early learning environment in phase 1 of study 2

b) International Development and Early Learning Assessment (IDELA) – it comprises of twenty-four items measuring socio-emotional development, motor development, emergent language and literacy, emergent maths and numeracy, executive functions and children's learning approaches (Pisani et al., 2018; Yousafzai et al., 2018; Wolf et al., 2017). The enumerators will administer IDELA in phase 2 of the study. Assessment will be conducted on children at the preschool setting or at home environment (for children who do not attend an ECE setting).

c) HOME early childhood scale. The HOME is a descriptive profile that leads to an assessment of a child's home environment. It measures the amount and quality of support and stimulation available to the child at home. Eight domains are measured: learning materials, language stimulation, physical environment, responsivity, academic stimulation, modelling, variety and acceptance. The scale will be shortened to meet project objectives and piloted to ensure cultural appropriateness of the items.

d) Qualitative Teacher Interview (QTI)- it elicits teachers' voices through a conversation about five key dimensions: (1) Context, (2) Teacher Experience, (3) Professional Development, (4) Learning opportunities and needs and (5) Quality of early learning environments. These dimensions have been previously identified as relevant when exploring quality learning environments in LMICs (Rao et al., 2019; Raikes et al., 2019).

The QTI will contextualise the MELE, IDELA and HOME early childhood scale data, as well as inform a Toolkit, which aims to further (1) Support the professional development of teachers to enhance children's learning; and (2) Enhance the quality of classroom interactions and the environment that can best support learning for children who are stunted, and (3) Gain a better understanding of the complexities of characterising quality early years settings.

Confidential: For Review Only

227 The reliability and validity of the measurements will be calculated and presented. The characterics of the methods and their acceptable validity data are presented in Table 2.

229 ***Interpretation of the measures***

230 A systematic method of translation and adaptation of the MELE, IDELA, HOME and QTI will be followed as previously described herein. Furthermore, the content MELE will be validated with external stakeholders involved in the implementation, administration and monitoring of ECE. Once the adapted measures are finalised, a back translation will be conducted. Discrepancies between the back-translated English version and the original English version should adapt to the cultural context and target population. However, the discrepancies will be revised if it is considered to depart from the intended meaning of the item.

237

238 **Table 2.**

239 *Age range, scoring procedures, administration time, reliability, validity and cross-cultural use of the measures selected for Study 2*

	**Age range**	**Scoring**	**Administration time**	**Reliability**	**Validity**	**Use in LMICs**
**IDELA**	3:6 to 6:6	Dichotomous (Correct, Incorrect), Continuous or Likert scales, based on direct observation	35 minutes	Inter-rater reliability [.70-.99] Internal Consistency [.66-.95]	Convergent validity: 0.33–0.61 Internal consistency: 0.66–0.95	India and Indonesia
**MELE**	Classroom-based observation	Dichotomous, continuous, Likert scales, or predefined answer options	120 minutes	Three factors: Health and Safety (α = .65), Materials and Activities (α = .87), Teacher-child interactions (α = .83)	Validated against policy regulations and curriculum for ECE	Tanzania
**HOME (early childhood scale)**	3:6 to 5:6	Dichotomous (Yes, No), based on direct observation	45 to 60 minutes	Inter-rater reliability [.70-.90]	Validity: 0.7	Bangladesh, Colombia and Mexico

Confidential: For Review Only

Analysis Plan

We will conduct both descriptive and inferential statistical analyses (i.e regression models with height as a continuous variable and Analysis of Covariance (ANCOVA)) to profile and compare the cognitive development and educational opportunities between siblings of the same household and children who are stunted and children who are not stunted in different settings. We will also use qualitative analysis to explore teachers' perceptions regarding positive educational environments. With our support country teams will create codebooks using Excel spreadsheets. We will implement thematic analysis (Silverman, 2016) by first conducting intra-case analysis (within country), followed by inter-case analysis (between countries) (Miles, Huberman and Saldaña, 2014).

Patient and public partnership strategy

The study proposal -including the methods used for assessing early learning and environment- was discussed with key stakeholders involved in the delivery of eary learning cirruculum in India, Indonesia and Senegal. Community engagement meetings were organised in the study communities to discuss the design, its benefits and the interpretation of its outcomes. Questions and answer sessions were included in the engagement plan to ensure questions are answered and there is adequate clarity around the purpose/intention of the research study. The study participants and public will be involved in the dessimation of the study findings.

Confidential: For Review Only

ETHICS AND DISSEMINATION

This study was approved by the institutional ethics committees of LSHTM, UCL Institute of Education and local country partner institutions in India, Indonesia and Senegal. The research is guided by the professional code of conduct of the British Educational Research Association (BERA) and British Psychological Society ethical guidelines. The research will be grounded in the ethical principles of confidentiality, informed consent, risk mitigation and 'do no harm'. The findings of the study will be presented in national and internation meetings, seminars, conferences and published in peer-reviewed journal. The study's data will be deposited in a public data repository.

Confidential: For Review Only

References

Bayley, N. & Aylward, G. P. (2019). Bayley Scales of Infant and Toddle Development, Fourth Edition. Pearson.

Black, M. M., Baqui, A. H., Zaman, K., Ake Persson, L., El Arifeen, S., Le, K., ... & Black, R. E. (2004). Iron and zinc supplementation promote motor development and exploratory behavior among Bangladeshi infants. *The American journal of clinical nutrition*, *80*(4), 903-910.

ECD Measures. (2018, April). MELQO Planning and Implementation Toolkit.

Fernandes, M. (2019). The Oxford Neurodevelopmental Assessment (OX-NDA) Manual v2.0.

Fernandes, M., Bassani, D., Albernaz, E., Bertoldi, A. D., Silveira, M.F, Matijsevich, A., Anselmi, L., Cruz, S., Hala, C. S., Tovo-Rodrigues, L., Nino Cruz, G. I., Metgud, D., Santos, I. S. (under review). Evaluation of the Oxford Neurodevelopment Assessment (OX-NDA) in 1-year-old Brazilian children: Findings from the 2015 Pelotas Birth Cohort Study.

Fernandes, M., Stein, A., Newton, C. R., Cheikh-Ismail, L., Kihara, M., Wulff, K., ... & International Fetal and Newborn Growth Consortium for the 21st Century (INTERGROWTH-21st). (2014). The INTERGROWTH-21st Project Neurodevelopment Package: a novel method for the multi-dimensional assessment of neurodevelopment in pre-school age children. *PloS one*, *9*(11), e113360.

Fernandes, M., Villar, J., Stein, A., Urias, E. S., Garza, C., Victora, C. G., ... & Kennedy, S. (2020). INTERGROWTH-21st Project international INTER-NDA standards for child development at 2 years of age: an international prospective population-based study. *BMJ open*, *10*(6), e035258.

Confidential: For Review Only

296 Iskandar, H. (2009). Realizing Quality Early Childhood Education and Parenting in Indonesia: Pitfalls and Strategies. In *International Conference on Early Childhood Education and Parenting* (pp. 1-9).

299 Jones, P. C., Pendergast, L. L., Schaefer, B. A., Rasheed, M., Svensen, E., Scharf, R., ... & MAL-ED Network Investigators. (2017). Measuring home environments across cultures: Invariance of the HOME scale across eight international sites from the MAL-ED study. *Journal of school psychology*, *64*, 109-127.

303 Miles, M. B., Huberman, A. M., & Saldaña, J. (2014). Qualitative data analysis: A methods sourcebook. 3rd. Thousand Oaks, CA: Sage.

305 Munoz-Chereau, Ang, L., Dockrell, J., Outhwaite, L., & Heffernan, C. (2021). Measuring early child development across low and middle-income countries: A systematic review. *Journal of Early Childhood Research*: ECR, 19(4), 443–470. https://doi.org/10.1177/1476718X211020031.

309 Murray-Kolb, L. E., Rasmussen, Z. A., Scharf, R. J., Rasheed, M. A., Svensen, E., Seidman, J. C., ... & Lang, D. (2014). The MAL-ED cohort study: methods and lessons learned when assessing early child development and caregiving mediators in infants and young children in 8 low-and middle-income countries. *Clinical Infectious Diseases*, *59*(suppl_4), S261-S272.

313 Nasir, M. T. M., Norimah, A. K., Hazizi, A. S., Nurliyana, A. R., Loh, S. H., & Suraya, I. (2012). Child feeding practices, food habits, anthropometric indicators and cognitive performance among preschoolers in Peninsular Malaysia. *Appetite*, *58*(2), 525-530.

316 Nurliyana, A. R., Shariff, Z. M., Taib, M. N. M., Gan, W. Y., & Tan, K. A. (2020). Early growth and home environment are associated with cognitive development in the first year of life of Malaysian infants. *Early human development*, *140*, 104890.

319 Perkins, J. M., Kim, R., Krishna, A., McGovern, M., Aguayo, V. M., & Subramanian, S. V. (2017). Understanding the association between stunting and child development in low- and

Confidential: For Review Only

middle-income countries: Next steps for research and intervention. Social Science & Medicine, 193, 101-109. doi:10.1016/j.socscimed.2017.09.039

Pisani, L., Borisova, I., & Dowd, A. J. (2018). Developing and validating the international development and early learning assessment (IDELA). *International Journal of Educational Research*, *91*, 1-15.

Raikes, A., Koziol, N., Davis, D., & Burton, A. (2020). Measuring quality of preprimary education in sub-Saharan Africa: Evaluation of the Measuring Early Learning Environments scale. *Early Childhood Research Quarterly*, *53*, 571-585.

Raikes, A., Sayre, R., Davis, D., Anderson, K., Hyson, M., Seminario, E., & Burton, A. (2019). The measuring early learning quality & outcomes initiative: purpose, process and results. *Early Years*, *39*(4), 360-375.

Rao, N., Ranganathan, N., Kaur, R., & Mukhopadhayay, R. (2021). Fostering equitable access to quality preschool education in India: challenges and opportunities. *International Journal of Child Care and Education Policy*, *15*(1), 1-22.

Salmon, L., & Dramani, L. (2015). Senegal: An Overview. In *Education in West Africa: Education Around the World* (Bloomsbury Academic, pp. 407–420).

Scharf, R. J., Rogawski, E. T., Murray-Kolb, L. E., Maphula, A., Svensen, E., Tofail, F., ... & DeBoer, M. D. (2018). Early childhood growth and cognitive outcomes: Findings from the MAL-ED study. *Maternal & child nutrition*, *14*(3), e12584.

Seurat, A. (2016). *Bilan des compétences des élèves à l'entrée au primaire au Sénégal*. UNICEF.

Silverman, D. (2016). *Qualitative research*. Sage Publications: London.

Singh, R., & Mukherjee, P. (2017). *Comparison of the Effects of Government and Private Preschool Education on the Developmental Outcomes of Children: Evidence From Young Lives India* (University of Oxford). Young Lives.

Confidential: For Review Only

United Nation Children's Fund. (2019). *A World Ready to Learn: Prioritizing quality early childhood education.* UNICEF.

Villar, J., Fernandes, M., Purwar, M., Staines-Urias, E., Di Nicola, P., Ismail, L. C., ... & Kennedy, S. (2019). Neurodevelopmental milestones and associated behaviours are similar among healthy children across diverse geographical locations. *Nature communications*, *10*(1), 1-10.

Wolf, S., Halpin, P., Yoshikawa, H., Dowd, A. J., Pisani, L., & Borisova, I. (2017). Measuring school readiness globally: Assessing the construct validity and measurement invariance of the International Development and Early Learning Assessment (IDELA) in Ethiopia. *Early Childhood Research Quarterly*, *41*, 21-36.

World Bank. (2020). *The Promise of Education in Indonesia.* World Bank.

Yousafzai, A. K., Rasheed, M. A., Rizvi, A., Shaheen, F., Ponguta, L. A., & Reyes, C. R. (2018). Effectiveness of a youth-led early childhood care and education programme in rural Pakistan: A cluster-randomised controlled trial. *PloS one*, *13*(12), e0208335.

Authors' contributions. Julie Dockrell and Lynn Ang designed Study 1 and 2; Jessica Massonnie and Julie Dockrell wrote the first draft of the paper; Bernardita Munoz-Chereau led the systematic review that drove the selection of measures for Study 1 and 2; Jessica Massonnie led the sampling plan for Study 2. Sylvia Fernandez Rao provided feedback regarding the implementation in India; Risatianti Kolopaking provided feedback regarding the implementation in Indonesia; Moustapha Ndiaye provided feedback regarding the implementation in Senegal; Claire Heffernan provided oversight of the study.

Funding statement. This work was supported by the UKRI GCRF Action Against Stunting Hub under Grant MR/S01313X/1.

Confidential: For Review Only

371 **Competing interests statement.** The authors declare no competing interest.

372

Confidential: For Review Only

BMJ Paediatrics Open

Measurement of cognition and profiling early learning environments in India, Indonesia, and Senegal: a UKRI GCRF Action Against Stunting Hub protocol paper

Journal:	*BMJ Paediatrics Open*
Manuscript ID	bmjpo-2022-001685.R1
Article Type:	Protocol
Date Submitted by the Author:	05-Dec-2022
Complete List of Authors:	Dockrell, Julie; UCL<br>Massonnié, Jessica; University of Portsmouth<br>Ang, Lynn; UCL<br>Munoz-Chereau, Bernardita; UCL, Learning and Leadership<br>Fernandez Rao, Sylvia; National Institute of Nutrition<br>Kolopaking, Risatianti; UIN Syarif Hidayatullah Jakarta<br>Ndiaye, Moustapha; Cheikh Anta Diop University of Dakar<br>Heffernan, Claire; UCL
Keywords:	Epidemiology, Growth, Nursing Care, Psychology

SCHOLARONE™
Manuscripts

I, the Submitting Author has the right to grant and does grant on behalf of all authors of the Work (as defined in the below author licence), an exclusive licence and/or a non-exclusive licence for contributions from authors who are: i) UK Crown employees; ii) where BMJ has agreed a CC-BY licence shall apply, and/or iii) in accordance with the terms applicable for US Federal Government officers or employees acting as part of their official duties; on a worldwide, perpetual, irrevocable, royalty-free basis to BMJ Publishing Group Ltd ("BMJ") its licensees and where the relevant Journal is co-owned by BMJ to the co-owners of the Journal, to publish the Work in this journal and any other BMJ products and to exploit all rights, as set out in our licence.

The Submitting Author accepts and understands that any supply made under these terms is made by BMJ to the Submitting Author unless you are acting as an employee on behalf of your employer or a postgraduate student of an affiliated institution which is paying any applicable article publishing charge ("APC") for Open Access articles. Where the Submitting Author wishes to make the Work available on an Open Access basis (and intends to pay the relevant APC), the terms of reuse of such Open Access shall be governed by a Creative Commons licence – details of these licences and which Creative Commons licence will apply to this Work are set out in our licence referred to above.

Other than as permitted in any relevant BMJ Author's Self Archiving Policies, I confirm this Work has not been accepted for publication elsewhere, is not being considered for publication elsewhere and does not duplicate material already published. I confirm all authors consent to publication of this Work and authorise the granting of this licence.

Confidential: For Review Only

Measurement of cognition and profiling early learning environments in India, Indonesia, and Senegal: a UKRI GCRF Action Against Stunting Hub protocol paper

Authors: Dockrell, Julie[1]; Massonnie, Jessica[2]; Ang, Lynn[3]; Munoz-Chereau, Bernardita[4]; Fernandez Rao, Sylvia[5]; Kolopaking, Risatianti[6]; Ndiaye, Moustapha[7]; Heffernan, Claire[8]

Affiliations: IOE, UCL's Faculty of Education and Society, UK [1 3 4]; University of Portsmouth, School of Education and Sociology (EDSOC)[2]; Behavioural Science Unit, Extension and Training Division, ICMR-National Institute of Nutrition, India[5]; London International Development Centre (LIDC) London School of Hygiene and Tropical Medicine, UCL Institute of Education, UK[8]; UIN Syarif Hidayatullah Jakarta, Indonesia[6]; Cheikh Anta Diop University Dakar, Senegal[7]

Corresponding author: Munoz-Chereau, Bernardita

Email: b.munozchereau@ucl.ac.uk

Key words: Cognition, Learning, Early Childhood Education, Children, Reducing Stunting, Early Development

Confidential: For Review Only

ABSTRACT

Introduction

Childhood stunting is associated with poorer child health, growth and development including diminished cognitive abilities. Mapping out the links between child stunting and Early Childhood Education and Development (ECED) is critical to increasing understanding of the causes and effects of childhood stunting, and for programme and policy development. The aim of this study is to investigate and compare the development and educational environments across India, Indonesia, and Senegal, and to identify the multifactorial drivers and impacts of childhood stunting to inform a new typology.

Methods and analysis

This current study is part of an interdisciplinary observational research study, where women are recruited during pregnancy and mother-infant pairs followed prospectively, up to 24 months after birth. Eight measures will be used to profile children's early development and learning environments in two sample cohorts: a) children aged 12 and 24 months born to the women recruited during pregnancy (i.e., 500 pregnant mothers per country); and b) a preschool case-control cohort of siblings from the main cohort aged between 3:6 to 5:6 years of age where anthropomorphic measures will be collected to assess degrees of stunting. Profiling of the development and learning environments in the countries will include both parent/caregiver self-reported and local staff (enumerators) direct assessments of children and settings.

Ethics and Dissemination

This study was approved by the institutional ethics committees of all partner institutions. In India, ICMR (Indian Council of Medical Research)-National Institute of Nutrition, Hyderabad; In Indonesia, Ethics Committee of the Faculty of Medicine, University of Indonesia; and in Senegal, National Ethics Committee for Scientific Research in Senegal.The

Confidential: For Review Only

findings of the study will be disseminated in national and international meetings, seminars, conferences and peer-reviewed journals.

Key message questions

**What is already known**	**What this study adds**	**How this study might affect research, practice and policy**
• Childhood stunting is associated with a range of developmental domains including cognition.<br>• Stunting co-occurs with challenging life circumstances, making it difficult to ascertain relationships between the impact of poor learning environments, cognition and stunting.<br>• There is a need to better understand the learning	• Enhances the knowledge base of measures of child cognitive development and learning environments for use in LMICs.<br>• Profiles the quality of learning environments in homes and in early years setting to better understand the links between stunting and child development in a range of settings.	• It enhances children's educational opportunities by building capacity in the study countries (India, Indonesia, and Senegal) to measure early development and learning environments in culturally and contextually appropriate ways.<br>• It provides a dataset to facilitate international comparisons and interpretation of other

Confidential: For Review Only

environments of children who are stunted, during and beyond the first two years of life.<br>• Childhood stunting is best considered on a continuum (child height for age Z score)<br>• Over the last decade a range of measures for assessing child development in Low- and Middle-Income Countries (LMIC) have been translated, adapted, and implemented.		data collected within the wider research programme.<br>• It provides an evidence-based approach to inform programme and policy development oriented to tackle childhood stunting.

Confidential: For Review Only

53

Confidential: For Review Only

INTRODUCTION

Childhood stunting is associated with a range of developmental domains including cognition, language, and motor development[1], where poorer HAZ (Height-for-Age Z scores) results are associated with lower scores in cognitive, language and motor development measures. Yet, causal pathways of impact are lacking. It is difficult to disentangle the impact of poorer learning environments and childhood growth patterns. Limitations in understanding have resulted from a lack of comparative measures across children, settings, and countries over time. Typically measures of cognition are designed for use in English speaking populations in high-income countries with limited attention to cultural and linguistic diversity. Furthermore, little work has focussed on assessing the educational and learning environments of children from areas where significant levels of childhood stunting are reported.

The UKRI GCRF Action Against Stunting Hub, with study sites in India, Indonesia, and Senegal, aims to apply a whole child approach to disrupt current thinking about the causes and impact of childhood stunting. The goal is to provide an integrated evidence base across the four inter-linked 'environments' of the child from the physical, home, and educational to the wider food environment. The cognition and education work stream underpins this effort by exploring the impact of HAZ on children's developmental trajectories and the ways in which learning environments can mitigate this impact. This study aims to assess the development and home learning environments of children aged between 12 and 24 months in India, Indonesia, and Senegal. Additionally, the study will also profile the early childhood education (ECE) environments attended by children between 3:6 and 5:6 years of age, and further compare the developmental profiles of children's HAZ in relation to growth limiting exposures[2].

The development of the protocol for this study is informed by a systematic review of reliable and valid measures of early development and learning environments in low- and middle-

Confidential: For Review Only

income countries[3], and by systematic profiling of the ECE systems in India, Indonesia, and Senegal. The study will examine the quality of learning environments in each country, the links between growth patterns and child development outcomes and delineate measures to enhance the educational opportunities for children.

Confidential: For Review Only

METHODS, SAMPLE AND ANALYSIS

This study is part of an interdisciplinary observational study to investigate the multiple factors implicated in child growth and development (child stunting). Prospective pregnancy cohorts were established in Senegal (Kaffrine), India (Hyderabad) and Indonesia (Lombok), where 500 women per country were recruited during pregnancy. The mothers and their infants will be followed up to 24 months of age to understand the contributions of multitude factors such as nutrition, gut health, epigenetics, food environment, food safety, hygiene, cognition, on childhood growth trajectories, where appropriate benchmarks of moderate and severe childhood stunting will be used in the analyses[4]. The sample size of the main population has been calculated based on statistical power and the frequency of stunting in the cohorts to be studied. The main calculation was based on the epigenetic work (see protocol by Jobarteh et al, titled Developing a 'whole child approach' to understanding and preventing child stunting: introducing the UKRI GCRF Action Against Stunting Hub). Based on the prevalence of stunting in the target populations (30-40%) we estimate that our strategy of random population sampling of $N = 500$ would yield the required numbers of 150-200 stunted children per study site.

The cognition and education workstream of the interdisciplinary research programme will focus on understanding early learning environments in the three study sites: India, Indonesia, and Senegal. Comparisons will allow the identification of similar and different patterns of relationships to further elucidate the links between poor childhood growth patterns and cognition, language, and motor development. The cognition and education research will be divided into two sub-studies:

Study 1

Confidential: For Review Only

This is a cross-sectional study designed to profile developmental skills and learning opportunities of infants at 12 – 24 months of age. Infants born to women recruited during pregnancy across communities in Senegal (Kaffrine), India (Hyderabad) and Indonesia (Lombok) will be enrolled in this study.

Data collection procedure

This study will involve direct assessment of children at 12 and 24 months of age by both local staff (enumerators) and parents/caregivers reports. The study will use specific direct child assessments and questionnaires to assess the different components of early child development. The measures include:

a) **Ox-NDA (12 months)**– the Oxford Neurodevelopment Assessment (OX-NDA) comprises of forty-one items measuring children's cognition, motricity, language, executive functions, positive behaviour, and negative behaviour[5,6]. The measure is designed to be used by trained enumerators, but no formal qualifications are required.

b) **Inter-NDA (24 months)**– the International Fetal and Newborn Growth Consortium for the 21st century Neurodevelopmental Assessment (Inter-NDA) comprises of thirty-nine items measuring cognition, motricity, language, positive behaviour, and negative behaviour[7-10]. The measure is designed to be used by trained enumerators, but no formal qualifications are required.

c) **Bayley IV Cognition scale (12 and 24 months)** – Following the test standardisation baseline measures will be established and children will progress through the assessment until they reach the discontinuation point t (starting and stopping rules are specified in the manual)[11]. This scale will be administered by appropriately qualified psychologists in each setting.

d) **HOME inventory (12 and 24 months)**– the infant and toddler HOME version used in previous studies examining the impact of stunting in LMIC (e.g., the MAL-ED

Confidential: For Review Only

study)[12-14] will be used. The HOME version comprises of forty-eight items and measures emotional and verbal responsivity from the caregiver, avoidance of restriction and punishment, promotion of child development by the caregiver, organisation of the physical and temporal environment, provision of appropriate play material, opportunities for variety in daily stimulation and cleanliness of child. The observation is scheduled at a time when the primary caregiver and the child are at home. All enumerators will be trained in the use of the HOME inventory.

The homes of study participants will be visited by local trained staff in each country and the questionnaire administered following a standard operating protocol for training and administration. The OX-NDA will be administered at twelve months of age and Inter-NDA will be administer at twenty-four months of age (i.e., one year later after OX-NDA). At both time points (12 and 24 months), observations of the home environment will be carried out concurrently using the HOME inventory, and fifty children (10% of the sample in each country) will be administered the Bayley IV cognition scale to cross-validate the Ox-NDA and Inter-NDA measures. To calculate the sample for the Bayley IV, we followed the results of Bridges and Holler[15] where data indicated that studies ought optimally to use 50 to 75 participants per cell. Our target is a minimum of 50 participant per cell (country by age group) with a total sample of 300 children for benchmarking.

Reliability and validity of the measurements will be calculated and the data for each measure presented. Table 1 reports validity and reliability of each measure obtained in previous relevant studies in LMIC[3]. Hence Table 1 is not exhaustive, but indicative of the evidence base for the inclusion of the measures.

Confidential: For Review Only

The rationale for choosing the measures was previously reported in a systematic literature review conducted at the beginning of the project[3]. Through that review we found suitable measures based on psychometric quality (validity and reliability), cultural adaptability and use in the field sites. Although we originally chose BSID-3 we decided to use BSID-4. This is a newer version of the BSID-3 capturing development in a more nuanced way, as items can be scored for mastery, emerging orr not present. It will be administered by experienced psychologists and overall, it will provide local teams with the experience and skills to apply it in the future.

Interpretation of the measures

A systematic process of translation, back translation and cultural adaptation will be followed for all measures to ensure that each questionnaire is valid and culturally appropriate for the study context[4]. The measures will be translated from English to the languages spoken at the study sites before use (India: Telegu and Hindi; Indonesia: Bahasa, Sasak; Senegal: French and Wolof). A panel of bilingual individuals will be convened to resolve expressions or concepts which may appear vague (i.e., either not understood or not clear) to ensure accurate translation of each question. Measures will then be back translated. All measures will be piloted in local settings. Regular meetings will take place with the Education and Cognition team members to ensure that the content, scoring options and materials depicted in the items are appropriate. Pilot data will be discussed, and onsite training will occur before the start of the main assessment period.

Table 1.

Comparison of the age range, scoring procedures, administration time, reliability, validity, and cross-cultural use of the measures obtained in previous studies and included in Study 1.

Confidential: For Review Only

	Age range	Scoring	Administration time	Reliability	Validity	Example of use in LMICs
**Ox-NDA**	10 to 14 months	Four-point Likert scale or three-points Likert scale, based on direct observation	20-30 minutes	Inter-rater reliability (k = .80-.96) Test-retest reliability (k = .85-.94)<br><br>Internal consistency >.70 for all subscales except language (α = .40) and negative behaviour (α = .22)	Moderate agreement between cognitive and motor domains of the OX-NDA and Bayley-III (ICCs: 0.63 and 0.68); satisfactory agreement for language (ICC: 0.30)	Brazil
**Inter-NDA**	22 to 30 months	Four-point Likert scale or three-points Likert scale, based on direct observation or caregiver report	15 minutes	Inter-rater reliability (k = .70) Test-retest reliability (k= .79)<br><br>Internal consistency: α > .80 (cognition, language, positive behaviour); α = .56 (negative behaviour)	Tested against Bayley III – better sensitivity and specificity for cognitive scale vs language scale	Brazil, India, and Kenya
**Bayley IV**	16 days to 24 months	Polytomous (not present, emerging, mastery) based on direct observation	Full scale: 50 min at 12 months 84 minutes at 24 months	Cognitive Scale: Test-Retest (*r* = 82) Internal consistency (split-half): *r* = .95	Less than 5-points discrepancy with WPPSI-IV and PDMS2	Bayley III has been used in Tanzania, Brazil, Malaysia, and Vietnam
**HOME**	0 to 3	Dichotomous (Yes, No) based on the observation	60 minutes	After removing Avoidance of Punishment,	Environmental Safety and Child Cleanliness	Columbia, Bangladesh, Mexico, Pakistan,

Confidential: For Review Only

	of the home environment, on caregiver's report, or either of them		three factors can be extracted (Emotional and Verbal Responsivity; Environmental Safety, Child Cleanliness) all with α > .70	correlate with crowding in the home.=	Jamaica, India, Nepal, Peru, Tanzania, Brazil, and South Africa

Notes. Bayley IV: Bayley Scales of Infant Development IV; Inter-NDA: International Fetal and Newborn Growth Consortium for the 21st Century Neurodevelopmental Assessment; OX-NDA: Oxford Neurodevelopmental Assessment at twelve months of age; PDMS-2: Primary Scale of Intelligence Second Edition; WPPSI-IV: Wechsler Preschool and Primary Scale of Intelligence Fourth Edition.

Confidential: For Review Only

Study 2

This second sub-study will compare the learning needs and educational opportunities of a preschool cohort of children aged between 3:6 and 5:6 years old using a case-control study design. The study will recruit older siblings of children who meet the age criterion across communities in Senegal (Kaffrine), India (Hyderabad) and Indonesia (Lombok). Measurements of childhood growth will be collected using standardised anthropomorphic measures. As with study 1 both a continuum of HAZ measures and cut-offs will be used to profile the population. This study will be conducted in two phases to provide a comprehensive understanding of the existing early years education setting and its link with child growth and development.

Phase 1: Profiling Early Learning Environments

A representative sample of early years settings will be selected in each study site following a systematic four-step process. Firstly, a documentary analysis will be used to establish the types of learning settings that exist in each study site and the proportion of children attending

201 the settings. Secondly, the number and types of settings in each study site will be referenced.
202 Thirdly, stratification variables will be outlined, including: the types of settings, source of
203 funding, targeted age group, locality (urban or rural), school size, class size, and the profile of
204 children attending the setting such as the socio-economic background, gender, ethnicity, and
205 religion [16-24]. Fourthly, a sample of settings will be selected in each study site according to
206 the stratification variables. The selection will include at least twenty percent of the existing
207 settings in the study site. In districts where there are 20 or fewer educational settings all will
208 be invited to participate in the study. The settings included in the sampling plan will be
209 visited in 2022.

210 *Phase 2: Comparisons of the learning needs and the environments of siblings*

211 Households of women recruited during pregnancy in the observational study will be
212 contacted for identification of households with children 3:6- to 5:6 years of age. Mothers in
213 eligible household will be asked if the identified child is enrolled in a preschool and the name
214 of the preschool. For children in preschool these settings will be contacted for participation in
215 the study. For children not in preschools child and learning environment measures will be
216 collected in the home. Children's cognitive performance will be profiled using the IDELA as
217 a robust and reliable global tool that measures children's early learning and development
218 (ages 3;5 to 6;0)[25-28].

219 Power calculation for inclusion of children was performed to determine the minimum sample
220 size needed to detect a small effect size ($f = .1$) in the analysis of the association between
221 height and child development (education and cognition) as reported in previous studies[29-
222 32]. The sample size was calculated at a power of 80% and an error probability of 5%,
223 considering the inclusion of seven covariates (age, gender, disability, family size, parental
224 education, parental income, and preschool attendance). The sample size calculations using
225 regression models with height as a continuous variable and Analysis of Covariance

Confidential: For Review Only

(ANCOVA) comparing stunted and not stunted children yielded a minimum sample size of 787 children. Thus, a target of 300 children will be recruited per country, a total of 900 children for the full study.

Data collection procedure

a) Local staff (enumerators) will be trained on all measures and piloting will take place with the enumerators. The early years settings will be visited during a normal preschool day to profile the learning environment. The days and times of observation will be randomly selected for each preschool using the preschool's schedule. In Phase 1 the MELE will be used to profile ECE, and teachers will be interviewed using a qualitative teacher interview (QTI). In Phase two children's profiles of development will be added and where children are not in ECEs the home learning environment will be profiled. Measuring Early Learning Environment (MELE) – contains fifty-two items grouped in five sections/grids: (i) basic classroom observation (e.g., number of children present), (ii) learning activities, (iii) classroom interactions and approaches to learning, (iv) classroom arrangement, space, and materials, (v) facilities and safety[17-20]. The MELE grid will be used to profile early learning environment in phase 1 of study 2.
b) International Development and Early Learning Assessment (IDELA) is comprised of twenty-four items measuring socio-emotional development, motor development, emergent language and literacy, emergent maths and numeracy, executive functions, and children's approaches to learning[25-28]. The enumerators will administer IDELA in phase 2 of the study. Assessment will be conducted with children at the preschool setting or in home environment (for children who do not attend an ECE setting).

Confidential: For Review Only

c) HOME early childhood scale. The HOME is a descriptive profile that leads to an assessment of a child's home environment. It measures the amount and quality of support and stimulation available to the child at home. Eight domains are measured: learning materials, language stimulation, physical environment, responsivity, academic stimulation, modelling, variety, and acceptance. The scale will be adapted to each local context and piloted to ensure cultural appropriateness of the items.

d) Qualitative Teacher Interview (QTI)- elicits teachers' voices through a conversation about five key dimensions: (1) Context, (2) Teacher Experience, (3) Professional Development, (4) Learning opportunities and needs and (5) Quality of early learning environments. These dimensions have been previously identified as relevant when exploring quality learning environments in LMICs[16-20].

The QTI will contextualise the MELE, IDELA and HOME early childhood scale data, as well as inform a Toolkit, which aims to further (1) Support the professional development of teachers to enhance children's learning; and (2) Enhance the quality of classroom interactions and the environment that can best support learning for children who are stunted, and (3) Gain a better understanding of the complexities of characterising quality early years settings.

The reliability and validity of the measurements will be calculated and presented. The characteristics of the methods and their reliability and validity reported in previous studies are presented in Table 2. The rationale for choosing the measures was previously reported in a systematic literature review conducted at the beginning of the project. Through that review we found suitable measures based on psychometric quality (validity and reliability), cultural adaptability[3] and use in the field sites.

Confidential: For Review Only

Interpretation of the measures

A systematic method of translation and adaptation of the MELE, IDELA, HOME and QTI will be followed as previously described herein. Furthermore, the content of MELE will be validated with external stakeholders involved in the implementation, administration, and monitoring of ECE. Once the adapted measures are finalised, a back translation will be conducted. Discrepancies between the back-translated English version and the original English version should adapt to the cultural context and target population. However, the discrepancies will be revised if it is considered to depart from the intended meaning of the item.

Table 2.

Comparison of the age range, scoring procedures, administration time, reliability, validity, and cross-cultural use of the measures obtained in previous studies and included in Study 2.

	**Age range**	**Scoring**	**Administration time**	**Reliability**	**Validity**	**Use in LMICs**
**IDELA**	3:6 to 6:6	Dichotomous (Correct, Incorrect), Continuous or Likert scales, based on direct observation	35 minutes	Inter-rater reliability [.70-.99] Internal Consistency [.66-.95]	Convergent validity: 0.33–0.61 Internal consistency: 0.66–0.95	India and Indonesia
**MELE**	Classroom-based observation	Dichotomous, continuous, Likert scales, or predefined answer options	120 minutes	Three factors: Health and Safety (α = .65), Materials and Activities (α = .87), Teacher-child interactions (α = .83)	Validated against policy regulations and curriculum for ECE	Tanzania
**HOME (early childhood scale)**	3:6 to 5:6	Dichotomous (Yes, No), based on direct observation	45 to 60 minutes	Inter-rater reliability [.70-.90]	Validity: 0.7	Bangladesh, Colombia and Mexico

Confidential: For Review Only

Notes. IDELA: International Development and Early Learning Assessment; MELE: Measuring Early Learning Environment; HOME (Early childhood scale): Home Observation for Measurement of the Environment.

Analysis Plan

We will conduct both descriptive and inferential statistical analyses. Given the large data sets and working across countries data cleaning and verification will be conducted by site. The first analyses will be done by country site and then, if appropriate conceptually and statistically, with comparisons across the three different sites to examine similarities and differences in patterns of relationships. Descriptive statistics will provide means (SDs) for child level IDELA and HOME by gender and age in months. MELE delivers a profile of scores to capture the learning environments. First, we will establish the profiles of early years provision across the study sites. This will provide a comparative benchmark with international data and for the ECE settings for the sibling cohort[17,18].

We plan three separate approaches to the analyses of child data. In the first instance we will use HAZ as a continuous variable in regression models to examine associations with developmental profiles controlling as appropriate for education settings. These analyses test the prediction that childhood preschool cognition is impacted by HAZ, controlling for learning environment, age, and gender. In a second set of analyses, we will use the WHO stunting criteria to compare children who are stunted and children who are not stunted. Finally, in order to account for the hierarchical nested nature of our data, we will explore relationships between the sibling's growth and cognition using multilevel modelling.

We will also use qualitative analysis to explore teachers' perceptions regarding positive educational environments. We will conduct Qualitative Teacher Interviews (QTI) with teachers

Confidential: For Review Only

and analyse them through thematic analysis. QTIs will be conducted face-to-face in the preschool settings, audio-recorded and transcribed. We will analyse the interview transcripts by developing a hybrid method to content analysis[33], which combines a deductive top-down approach (that explores the presence of themes informed by categories drawn from previous knowledge of the literature and themes derived from our research questions) with an inductive bottom-up approach (what participants said). The deductive coding will consist of interrogating the data using categories informed by the interview schedule (such as "context" and "teacher experience").

More precisely, the deductive coding will explore the following aspects presented in Table 3.

Table 3.

Codes to be used in the deductive coding of QTI

QTI section/categories	QTI questions	Codes
	What is the age range of the children enrolled in your classroom?	Age range
	What proportion of children are aged 3-5?	Proportion
	Is children's height and weight measured? If yes: How is it measured? How often? How is it stored? If no: Do you access to these measures? Who provides these measures?	Height and weight
	Is food served at the programme (either a meal or snack provided by the programme or children bring from home)? If yes: What meals or snacks are served? (Snack served only, Lunch served only, Snacks and lunch are served), Who provides the food? (The programme provides all food, The programme provides some food and parents provide some, Parents provide all food), Is there a daily menu? In a normal week, how many days is a meal served? (None, 1-3 4+), If a meal is served less than every day, please indicate the reason why: Holiday, Ration not available, Other (specify)	Food
	Is water served at the programme? If yes: Where does the water come from? Who provides the water? How do children drink it? (i.e., cups, bottles)	Water
Context	How COVID has affected the organisation of learning (i.e., conditions, who is attending)?	Covid

Confidential: For Review Only

	What is your main role in the [centre/setting]?	Main role
	How many years have you worked in this pre-primary center?	Years in the setting
	How many years have you been a teacher overall (for any grade)?	Overall
	What is the highest qualification level you have completed?	Highest qualification
	Did you need to complete a qualification or training to become a teacher? If yes: What was it?	Requirements
	Why did you choose to become an early years teacher?	Reason
Teacher Experience	If you could turn back time, would you choose again to become a teacher? Why?	Choice
Professional development	What training opportunities are there in your setting?	Professional opportunities
Professional development	If you were offered training, what would you find useful?	Useful training
Children learning experiences and opportunities	Please describe a normal working day with the children	Routine
	What activities are routinely provided?	Activities
	What do you think children enjoy most about coming here?	Enjoy
	What do you see as most important in children's education?	Priority
	When a child starts pre-school here, what can they normally do on their own?	Can do
	How do you know if a child needs more support with their learning?	Support identified
	How do you support their learning?	Support provided
	Are there any targets or goals that you use to guide teaching and learning? If yes: What are these targets? Who defines these targets? Are they evaluated? How?	Targets
Children learning experiences and opportunities	Do you keep children's portfolios? If yes: How does it work? Who decides what goes on the portfolio? Who has access to the portfolio?	Portfolios
	According to your view, what are the main opportunities/strengths in the learning environment you work in?	Opportunities
	What support would improve children's learning opportunities? (I.e more materials, more training, community support)	Needs
Quality early learning environment	When you think about the best preschool: How is teaching delivered? How the environment looks like? how is it the space set up? What materials are available? What interactions are promoted?	Ideal scenario

Confidential: For Review Only

	How would you describe the main similarities between these pictures and your setting?	Similarities
	How would you describe the main differences between these pictures and your setting?	Differences
Emergent	Is there anything else you would like to add?	Emergent

The deductive coding will be followed by inductive coding where we will assign meaning to segments of text using categories (for example ‘challenges’ or ‘support’) that might appear in the interview transcripts. Both coding procedures will break down the transcriptions into smaller pieces of information and compare the pieces for similarities and differences before regrouping them under themes and categories[34]. We will create coding schemes using Excel spreadsheets. Interviews will be analysed into one coding scheme per country. We will implement intra-case analysis (within country), followed by inter-case analysis (between countries)[35]. We will do this by taking an iterative process of categorizing and connecting data in order to understand how the data relate and interact within and across contexts.

The QTI thematic analysis will be integrated with other measures (MELE and IDELA) to gain a better understanding of a) the quality of early years settings from the perspectives of teachers and adults involved in supporting children’s learning and b) the specific cultural context of what makes a positive learning environment according to each setting.

Patient and public partnership strategy

The study proposal, including the methods used for assessing early learning and environment,was discussed with key stakeholders involved in the delivery of early learning curriculum in India, Indonesia, and Senegal. Community engagement meetings were organised in the study communities to discuss the design, its benefits, and the interpretation of its outcomes. Questions and answer sessions were delivered in the engagement plan to ensure questions are answered and there is adequate clarity around the purpose/intention of

Confidential: For Review Only

346 the research study. The study participants and public will be involved in the dissemination of

347 the study findings.

348

Confidential: For Review Only

Ethics and Dissemination

As a research team, we are committed to the principles of fair, equitable and ethical research partnerships and ensuring that the research is undertaken to the highest professional standards of ethics and integrity. This study was approved by the institutional ethics committees of all partner institutions. In India, ICMR (Indian Council of Medical Research)-National Institute of Nutrition, Hyderabad; In Indonesia, Ethics Committee of the Faculty of Medicine, University of Indonesia; and in Senegal, National Ethics Committee for Scientific Research in Senegal. The research will be grounded in the ethical principles of confidentiality, informed consent, risk mitigation and 'do no harm.' We are guided by the professional code of conduct specific to each discipline as set out in the British Educational Research Association and British Psychological Society ethical guidelines. The findings of the study will be presented in national and international meetings, seminars, conferences and published in peer-reviewed journals. The study's data will be deposited in a public data repository.

Confidential: For Review Only

References

(1) Perkins JM, Kim R, Krishna A et al. Understanding the association between stunting and child development in low-and middle-income countries: Next steps for research and intervention. *Soc. Sci. Med* 2017;193:101-9.

(2) Perumal N, Bassani DG, Roth DE. Use and misuse of stunting as a measure of child health. *Nutr. J* 2018;148(3):311-5.

(3) Munoz-Chereau B, Ang L, Dockrell J et al. Measuring early child development across low and middle-income countries: A systematic review. *Early Child. Res. Q.* 2021;19(4):443-70.

(4) Fernald LC, Prado E, Kariger P et al. A toolkit for measuring early childhood development in low and middle-income countries. World Bank 2017.

(5) Banerjee K, Dwivedi LK. Disparity in childhood stunting in India: Relative importance of community-level nutrition and sanitary practices. *PloS* one 2020.1;15-9

(6) Fernandes M. The Oxford Neurodevelopmental Assessment (OX-NDA) 2019. Manual v2.0.

(7) Fernandes M, Bassani D, Albernaz E et al. Evaluation of the Oxford Neurodevelopment Assessment (OX-NDA) in 1-year-old Brazilian children: Findings from the 2015 Pelotas Birth Cohort Study (under review).

(8) Fernandes M, Stein A, Newton CR et al. The INTERGROWTH-21st Project Neurodevelopment Package: a novel method for the multi-dimensional assessment of neurodevelopment in pre-school age children. *PloS one* 2014 25;9-11

(9) Fernandes M, Villar J, Stein A et al. INTERGROWTH-21st Project international INTER-NDA standards for child development at 2 years of age: an international prospective population-based study. *BMJ open* 2020 Jun 1;10-6.

Confidential: For Review Only

(10) Villar J, Fernandes M, Purwar et al. Neurodevelopmental milestones and associated behaviours are similar among healthy children across diverse geographical locations. *Nat. Commun* 201910;1-10.

(11) Bayley N, Aylward GP. Bayley Scales of Infant and Toddler Development fourth edition (Bayley-4). Bloomington, MN: NCS Pearson. 2019.

(12) Black MM, Baqui AH, Zaman K et al. Iron and zinc supplementation promote motor development and exploratory behavior among Bangladeshi infants. *AJCN* 2004;80(4):903-10.

(13) Jones PC, Pendergast LL, Schaefer BA et al. Measuring home environments across cultures: Invariance of the HOME scale across eight international sites from the MAL-ED study. *J. Sch. Psychol* 2017; 64:109-27.

(14) Bridges AJ, Holler KA. How many is enough? Determining optimal sample sizes for normative studies in pediatric neuropsychology. *Child Neuropsychol* 2007;13(6):528-38.

(15) Murray-Kolb LE, Rasmussen ZA, Scharf RJ et al. The MAL-ED cohort study: methods and lessons learned when assessing early child development and caregiving mediators in infants and young children in 8 low-and middle-income countries. *Clin. Infect. Dis*2014;59:261-72.

(16) Iskandar H. Realizing Quality Early Childhood Education and Parenting in Indonesia: Pitfalls and Strategies. InInternational Conference on Early Childhood Education and Parenting 2019 (ECEP 2019) 2020 Aug 10 (pp. 1-9). Atlantis Press.

(17) Rao N, Richards B, Sun J et al. Early childhood education and child development in four countries in East Asia and the Pacific. *Early Child. Res. Q.* 2019; 47:169-81.

(18) Raikes A, Sayre R, Davis D et al. The measuring early learning quality & outcomes initiative: purpose, process and results. *Early Years* 2019; 39(4):360-75.

Confidential: For Review Only

(19) Raikes A, Koziol N, Davis D et al. Measuring quality of preprimary education in sub-Saharan Africa: Evaluation of the Measuring Early Learning Environments scale. *Early Child. Res.Q.* 2020;53:571-85.

(20) Rao N, Ranganathan N, Kaur R et al. Fostering equitable access to quality preschool education in India: challenges and opportunities. *Int. J. Child Care Educ. Policy* 2021;15(1):1-22.

(21) Salmon L, Dramani L. Senegal: An overview. Education in West Africa. 2015;29(56.7):407-420.

(22) Seurat A. *Bilan des compétences des élèves à l'entrée au primaire au Sénégal*. 2016. UNICEF.

(23) Singh R, Mukherjee P. Comparison of the Effects of Government and Private Preschool Education on the Developmental Outcomes of Children: Evidence From Young Lives India.Young Lives 2017. University of Oxford.

(24) UNICEF. A World Ready to Learn: Prioritizing Quality Early Childhood Education: UNICEF. New York, April. 2019.

(25) Scharf RJ, Rogawski ET, Murray,Ä et al. Early childhood growth and cognitive outcomes: Findings from the MAL-ED study. *Maternal & Child nutrition* 2018;14(3):125-84.

(26) Pisani L, Borisova I, Dowd AJ. Developing and validating the international development and early learning assessment (IDELA). International Journal of Educational Research 2018;91:1-5.

(27) Yousafzai AK, Rasheed MA, Rizvi A et al. Effectiveness of a youth-led early childhood care and education programme in rural Pakistan: A cluster-randomised controlled trial. *PloS one* 2018;13:1-14.

(28) Wolf S, Halpin P, Yoshikawa H et al. Measuring school readiness globally: Assessing the construct validity and measurement invariance of the International

Confidential: For Review Only

Development and Early Learning Assessment (IDELA) in Ethiopia. *Early Child. Res. Q.*2017;41:21-36.

(29) Afkar R, Prakosa PW, Couslon J et al. The Promise of Education in Indonesia. World Bank 2020.

(30) Nasir MT, Norimah AK, Hazizi AS et al. Child feeding practices, food habits, anthropometric indicators and cognitive performance among preschoolers in Peninsular Malaysia. *Appetite* 2012;58(2):525-30.

(31) Nurliyana AR, Shariff ZM, Taib MN et al. Early growth and home environment are associated with cognitive development in the first year of life of Malaysian infants. *Early human development* 2020;140:1048-90.

(32) Koshy B, Srinivasan M, Gopalakrishnan S at al. Are early childhood stunting and catch-up growth associated with school age cognition?—Evidence from an Indian birth cohort. PloS one 2022;17(3):1-14.

(33) Chandra Y, Shang L. Inductive coding. In Qualitative research using R: A systematic approach 2019 (pp. 91-106). Springer, Singapore.

(34) Silverman, D. (2016). Qualitative research. Sage Publications: London.

(35) Miles, M. B., Huberman, A. M., & Saldaña, J. (2014). Qualitative data analysis: A methods sourcebook. 3rd. Thousand Oaks, CA: Sage.

Authors' contributions. Julie Dockrell and Lynn Ang designed Study 1 and 2; Jessica Massonnie and Julie Dockrell wrote the first draft of the paper; Bernardita Munoz-Chereau led the systematic review that drove the selection of measures for Study 1 and 2; Jessica Massonnie led the sampling plan for Study 2. Sylvia Fernandez Rao provided feedback regarding the implementation in India; Risatianti Kolopaking provided feedback regarding

Confidential: For Review Only

the implementation in Indonesia; Moustapha Ndiaye provided feedback regarding the implementation in Senegal; Claire Heffernan provided oversight of the study.

Funding statement. This work was supported by the UKRI GCRF Action Against Stunting Hub under Grant MR/S01313X/1.

Competing interests statement. The authors declare no competing interest.

Confidential: For Review Only
